# An interactive soft robotic hand-task training system with wireless task boards and daily objects on post-stroke rehabilitation

**DOI:** 10.1017/wtc.2024.10

**Published:** 2025-02-03

**Authors:** Xiangqian Shi, Chengyu Yang, Pak Chung Lee, Disheng Xie, Zhongping Ye, Zheng Li, Raymond Kai-yu Tong

**Affiliations:** 1 Department of Biomedical Engineering, The Chinese University of Hong Kong, Hong Kong SAR, China; 2 Hopebotic Limited, Hong Kong SAR, China; 3 Department of Surgery, The Chinese University of Hong Kong, Hong Kong SAR, China

**Keywords:** stroke rehabilitation, soft robotics, robot-assisted therapy, task-oriented training, daily task assistance

## Abstract

We have developed an interactive system comprising a soft wearable robot hand and a wireless task board, facilitating the interaction between the hand and regular daily objects for task-oriented training in stroke rehabilitation. A ring-reinforced soft actuator (RSA) to accommodate different hand sizes and enable flexion and extension movements was introduced in this paper. Individually controlled finger actuators assist stroke patients during various grasping tasks. A wireless task board was developed to support the training, allowing for the placement of training objects and seamless interaction with the soft robotic hand. Evaluation with seven stroke subjects shows significant improvements in upper limb functions (FMA), hand-motor abilities (ARAT, BBT), and maximum grip strengths after 20 sessions of this task-oriented training. These improvements were observed to persist for at least 3 months post-training. The results demonstrate its potential to enhance stroke rehabilitation and promote hand-motor recovery. This lightweight, user-friendly interactive system facilitates frequent hand practice and easily integrates into regular rehabilitation therapy routines.

## I. Introduction

A prevalent symptom among stroke survivors is motor deficits (Basteris et al., 2014). This motor impairment significantly impedes stroke patients’ ability to perform activities of daily living (ADL) (Timmermans et al., 2010), subsequently impacting their quality of life (QOL). Notably, approximately 80 percent of stroke survivors encounter post-stroke deficits in upper extremity (UE) motor performance, with approximately half of them facing challenges in performing ADLs (Kwakkel et al., 2003; Duncan et al., 2003).

Several stroke survivors who underwent a UE rehabilitation program demonstrated significant recovery of proximal motor functions at the shoulder and elbow joints. However, their progress in regaining hand and wrist joint functionality remained limited (Jonkman et al., 1998). Hand functions, especially for finger extension, are vital in numerous daily tasks. Nevertheless, developing an effective training device for rehabilitating dexterous hand functions has posed a considerable challenge.

One potential approach to address these challenges is the development of rehabilitation robotic devices (Maciejasz et al., 2014; Song et al., 2008; Wolbrecht et al., 2018). In recent decades, hand rehabilitation devices have been introduced to aid stroke survivors in recovering hand functionality (Susanto et al., 2015; Ho et al., n.d.; Hu et al., 2009). However, these devices have limitations, notably their bulky and heavy nature resulting from using rigid components like linear motors and rigid linkages connecting the fingers to the motors (Dellon and Matsuoka, 2007). Consequently, stroke patients often face difficulty raising their paralyzed arms and performing functional tasks (Tong et al., 2010). Moreover, these robotic hands seldom account for the individual’s specific hand conditions, such as finger length, palm width, and joint stiffness.

Our prior research presented a 3D-printed soft robotic hand that utilized the soft-elastic composite actuator (Heung et al., 2019; Heung et al., 2020; Shi et al., 2021; Tang et al., 2022; Shi et al., 2020). Building upon this foundation, we have further advanced our work by developing an updated version of the interactive soft wearable robotic hand specifically designed for task-oriented hand-functional training. The new version of the pneumatic actuator in this version offers enhanced actuation torque, while a novel wireless interactive task training board has been designed and seamlessly integrated with the robotic hand. This innovative solution demonstrates our commitment to addressing the unique requirements of stroke rehabilitation, offering a more personalized and interactive approach to therapy.

## II. Interactive soft robotic hand system

### A. Wireless task training board

The overall set of the hand-task training system is shown in Figure 2. We developed a wireless task training board to achieve interactive assistance for the soft robotic hand. The task board is equipped with an array of Hall effect sensors, and a magnetic component is affixed inside the soft robotic hand.

The wireless task board utilizes magnetic field intensity detection to determine the distance between the soft robotic hand and itself. Once a predetermined threshold is exceeded, the wireless communication module within the task board transmits instructions to the controller, triggering pre-set gesture assistance by the soft robotic hand. To achieve diverse hand functions during task-oriented training, such as five-finger grasps (Cylindrical/Spherical power), three-finger grasp (Tripod pinch), two-finger grasp (Tip/Lateral pinch), and finger extension, the soft robotic hand can connect with multiple task boards. This enables the realization of various task training designs by incorporating different task boards with distinct hand-function interactions.

Figure 3 provides a concise example of a moving objects task, showcasing the functionality of the task boards. The black task board is configured for spherical power grasp assistance, while the white task board is set for hand-opening assistance. In this scenario, a stroke patient wearing the soft robotic hand is instructed to transfer balls (d = 80 mm) from the black task board to the basket placed on the white task board. The training process involves three main steps:

- The patient approaches the black task board with their affected hand, aiming to grasp the ball. As the hand nears the board, the magnetic field intensity reaches a specific threshold, triggering the soft robotic hand to provide grasping assistance. Once the grasp is established, the system allows a brief pause to allow the patient to adjust their grip and lift the hand away from the black task board.
- As the patient’s hand moves away from the black task board and enters the task’s execution phase, the soft robotic hand continues to support and assist in maintaining the grasp, ensuring a stable hand posture throughout the task.
- When the patient’s hand approaches the basket on the white task board, the same detection method as the black task board is employed to assess the distance between the hand and itself. Once the proximity reaches a certain threshold, the soft robotic hand is triggered to provide hand-opening assistance, aiding the patient in releasing the ball into the basket.

### B. Actuator design

**Figure 1.**
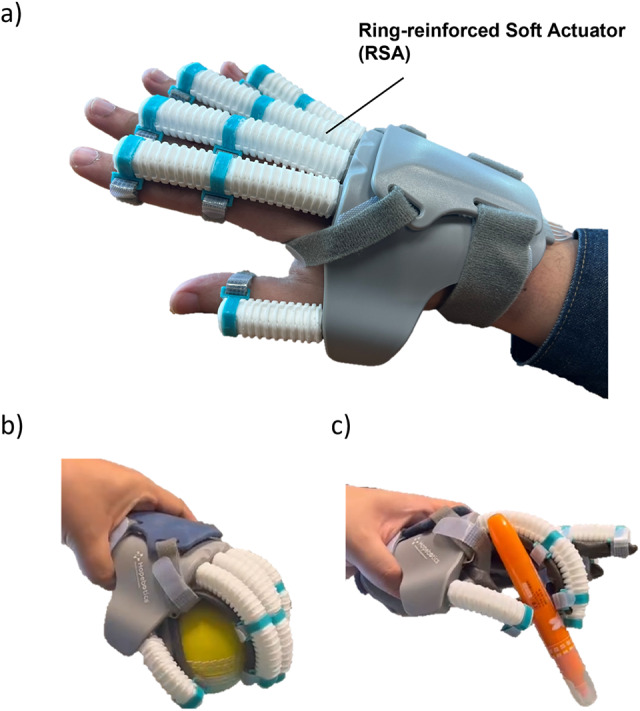
(a) Prototype of the soft wearable robotic hand, (b) spherical grasp a ball by robotic hand, and (c) tripod pinch a marker pen by robotic hand.

Our previous studies described the soft-elastic composite actuator (SECA) utilized in the early version of the soft robotic hand (Heung et al., 2019; Heung et al., 2020; Tang et al., 2021). However, the original design possesses certain limitations. First, wrapping the fibers is time-consuming. Second, prolonged usage tends to loosen the fibers, rendering the actuator susceptible and significantly impacting its performance. To improve the performance of the soft actuator, we have introduced a novel design known as the RSA, shown in Figure 1. The RSA comprises several key components, including an actuator body made of elastomeric materials, plastic ring constraints, and a torque-compensating layer. The operational principle of the RSA closely resembles that of the originally designed SECA. Utilizing a single pressurizing source, the torque-compensating layer enables finger flexion and extension within the same actuator unit.

The elastomeric body and soft bottom pad of the RSA are 3D 3D-printed using ACEO Silicone GP Shore A 30 (WACKER Chemie AG), which can offer sufficient elongation (450%) and tensile strength (6 MPa). Since the distal interphalangeal (DIP) joints only play a limited role of 15% of functional gripping (Leibovic and Bowers, 1994), the RSA only covers the two joints of each finger: proximal interphalangeal (PIP) and metacarpophalangeal (MCP) joints. The center of each segment is aligned with a corresponding finger joint. The torque-compensating layer was placed between the elastomeric body and the soft bottom pad. When pressure is applied, the ring constraints effectively prevent radial expansion, while the torque-compensating layer restricts axial elongation at the bottom, leading to a bending motion (Figure 1b and c). Conversely, during depressurization, the torque-compensating layer enables the RSA to return smoothly to its original undeformed position.

### C. Actuator performance

Previous studies show the significance of achieving adequate actuation torque (>0.2 Nm) (Bützer et al., 2021) and range of motion (>120°) (Polygerinos et al., 2015) when designing a rehabilitation robotic hand. In this study, we focused on assessing the performance of the RSA. To ensure comparability with human finger sizes (Peters et al., 2002), the RSA used in our tests had dimensions of 10 cm in length, 1.8 cm in width, and height proportional to human fingers. The force exerted at the fingertip varies based on the lever arm, which changes with different finger positions. Consequently, we evaluated the fingertip force across various overall finger flexion angles (0°, 40°, 80°, and 120°) for increasing input air pressure (from 0 kPa to 300 kPa, with an interval of 25 kPa). To measure the input force, we employed a compression load cell (FC22, Measurement Specialties, U.S.A.). The proximal end of the RSA was clamped to establish a vertical orientation (Figure 4a. The maximum input pressure applied to the RSA was restricted to 300 kPa. Remarkably, the results demonstrated that the RSA achieved an actuation torque of 0.3 Nm when the overall finger flexion angle reached 120° (Figure 4b). No wall rupture or air leakage was observed throughout the bending angle measurement process.

### D. Soft robotic hand module

The soft robotic hand module comprises five RSAs seamlessly integrated into a plastic hand brace. To ensure stability and comfort, Velcro straps are utilized to secure the wrist, palm, and finger joints in conjunction with the soft robotic hand (refer to Figure 1a). Both the size of the hand brace and the length of the RSAs are custom-designed and 3D printed to fit the user’s specific hand dimensions accurately. The total weight of the soft robotic hand is 150 grams, which is lightweight enough to avoid placing any additional burden on the impaired hand, thus preserving natural hand movement capabilities.

**Figure 2.**
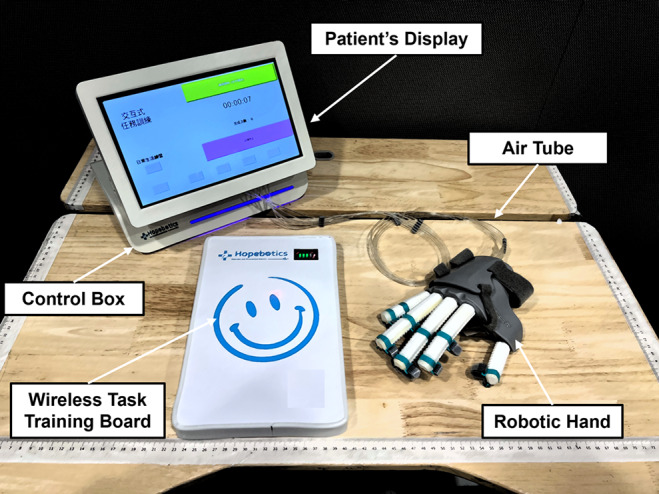
Full set of interactive soft robotic hand-task training systems.

The interactive pneumatic control system is built to control the soft robotic hand (Figure 2). Air tubes connected the hand to the control box, and five three-way pneumatic solenoid values controlled the actuators individually. Two small air pumps were installed inside the control box, which could provide compressed air pressure up to 320 kPa. An embedded controller is built to control the on–off solenoid valves by regulating the output PWM signal. Pressure sensors (XGZP6847, CF Sensors, Korea) monitor the air pressure inside multiple RSAs to determine whether the RSAs are fully inflated and prevent over-pressurization.

**Figure 3.**
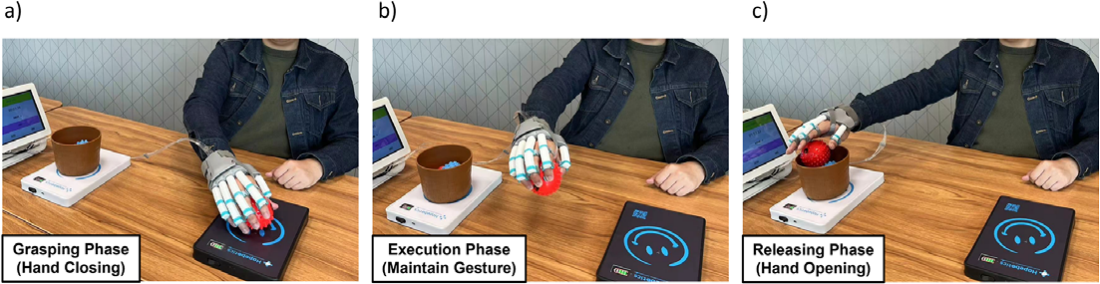
Example of task training process for moving objects task (ball, d = 80 mm). (a) Patient wearing a soft robotic hand approaches the task board (black), and activates interactive grasp mode for ball grasping, (b) Patient’s hand leaves the task board (black), soft robotic hand maintains grasping assistance during the task execution phase.

**Figure 4.**
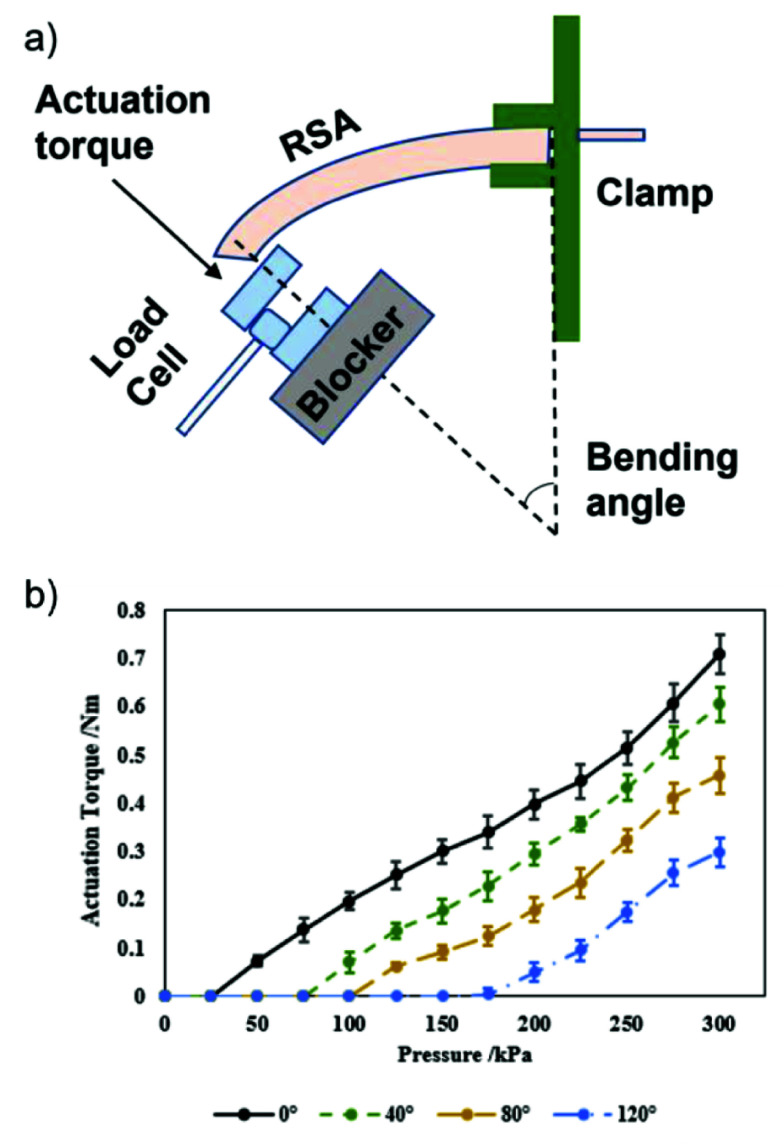
(a) Measurement setup for fingertip forces in the function of the finger flexion angle, (b) actuation torque (mean and SD.) in the function of the input pressure and the overall finger flexion angle.

## III. Evaluation by stroke survivors

### A. Stroke subjects

A total of seven chronic stroke subjects were enlisted to assess the effectiveness of the soft robotic hand in conjunction with task-oriented hand-functional training. Each subject participated in 20 sessions of rehabilitation training. The demographic information of the recruited subjects can be found in Table 1.

**Table 1.** Demographic information

Subject	Gender	Age	Stroke type	Affected side	Stroke onset (years)
1	M	47	Isch.	Right	2.8
2	M	61	Isch.	Right	5.5
3	M	65	Hemo.	Left	6.4
4	F	71	Hemo.	Right	3.7
5	F	50	Isch.	Left	4.7
6	M	34	Isch.	Right	1.6
7	M	68	Hemo.	Left	2.2

Female (F); Male (M); Hemorrhage (Hemo); and Ischemia (Isch).

### B. Task-oriented hand-functional training

In this evaluation, each subject was required to attend 20 training sessions, with an intensity of about 3–5 sessions per week. Clinical outcome measures were assessed before and after the 20 sessions. During each session, the total task execution cycles were recorded and shown on the patient’s display.

**Figure 5.**
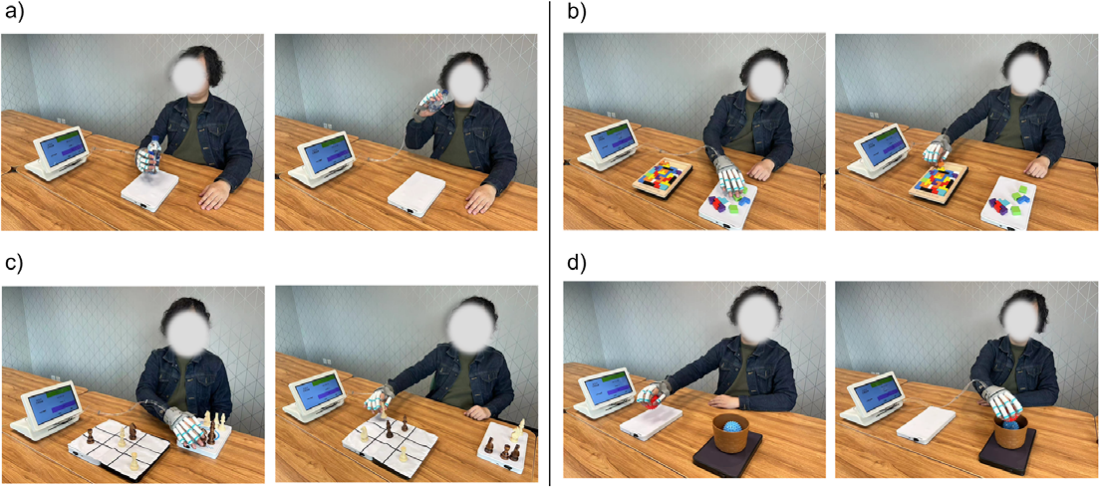
Four different tasks: (a) drinking task (bottle full of water, 330 mL), (b) puzzle task (small blocks with diameters around 2–3 cm), (c) chess game (standard wooden chess pieces), and (d) moving task (Ball with a diameter of 80 mm).

During the training sessions, stroke subjects participated in four sets of hand-functional training tasks facilitated by the soft robotic hand. Each training session lasted for 10 min. The training objects were positioned on a task board, with the number of task boards utilized depending on the task requirements (refer to Figure 5). The training encompassed the following tasks:

- Drinking task: One task board with both hand cylindrical grasping (five fingers) and opening assistance was utilized in this task. This task involved the subject gripping a water bottle from the task board (cylindrical grasp assistance), raising the arm, simulating a drinking motion by bringing the water bottle to the mouth, returning the water bottle to the task board, and triggering the hand-opening action.
- Puzzle task: Two task boards with tip pinching (two fingers) and hand-opening were utilized in this task. The therapist placed small object fragments on one task board and the puzzle base on another. The subjects were required to pick up the small objects from the first task board (tip pinch assistance) and attempt to place them in the puzzle base on the other side, aiming to form a complete rectangle.
- Chess game: Three task boards with tripod pinching (three fingers) and hand-opening (use two boards to enlarge the surface area) were utilized in this task. In this task, the subject played chess against the therapist. The subjects picked a chess piece of a particular color from a task board and placed it on a chess cloth (comprising two additional task boards). The objective was to be the first to align three pieces of the same color in a straight line.
- Moving task: Two task boards with spherical grasping (five fingers) and hand-opening were utilized in this task. Subjects were tasked with grasping a ball from one task board and transferring it to a basket positioned on another.

### C. Outcome measurements

Multiple clinical assessments were utilized to evaluate motor improvement resulting from the robot-assisted task-oriented hand-functional training immediately after the training and during a follow-up after 3 months. These assessments included the Fugl–Meyer assessment (FMA), action research arm test (ARAT), box and block test (BBT), and modified Ashworth scale (MAS). The FMA evaluates voluntary motor functions of the upper limb and consists of two separate measurements: the shoulder and elbow section (S/E) and the wrist and hand section (W/H). The ARAT and BBT measure hand functions by assessing daily activity tasks. Furthermore, the maximum grip strength (MGS) and maximum pinch strength (MPS) assessments were employed to evaluate improvements in hand functionality. Paired t-tests were applied to examine the changes compared with the pre-training scores.

The evaluation trial consisted of assessments also conducted before training, immediately after training, and during a 3-month follow-up. Each subject was instructed to complete the puzzle task twice within 3 min. In the first trial, subjects utilized the soft robotic hand, while in the second trial, they performed the task without it. The number of successfully grasped small objects transferred into the puzzle base was recorded separately for each trial.

## IV. Results

Table 2 presents a comprehensive overview of the training outcomes before and after the 20-session task-oriented hand-functional training. The results reveal significant motor improvements across various clinical assessments, including Fugl-Meyer Assessment (FMA), FMA-W/H (wrist and hand section), FMA-S/E (shoulder and elbow section), action research arm test (ARAT), box and block test (BBT), and maximum grip strength (MGS). The notable enhancements in ARAT and BBT scores indicate improved motor recovery in hand and finger functions. Moreover, the increased FMA scores suggest overall motor improvement in the entire upper limb following the training. The improvement in MGS and MPS signifies enhanced capabilities in performing practical hand-grasp functions.

**Table 2.** Statistical summary

Outcome	Mean ± SD			t-test	
(max. score)	Pre (1)	Post (2)	Follow-up (3)	(2)–(1)	(3)–(1)
FMA (66)	19.8 ± 8.2	26.8 ± 8.7	28.0 ± 9.9	.003*	.032*
W/H (24)	5.2 ± 3.9	8.8 ± 6.6	9.8 ± 8.0	.009	.046*
S/E (36)	12.5 ± 6.2	15.5 ± 6.6	15.5 ± 5.9	<.001*	.009*
ARAT (57)	15.5 ± 5.1	21.3 ± 6.7	21.0 ± 5.9	.002*	.007*
BBT (60)	2.2 ± 0.6	4.0 ± 2.2	4.2 ± 2.	.048*	.049*
MGS	4.4 ± 2.2	7.2 ± 2.6	7.0 ± 2.4	.006*	.008*
MPS	0.4 ± 0.6	1.3 ± 1.1	1.4 ± 1.0	.102	.097

* Mean value changes with statistical significances (p < .05)

**Figure 6.**
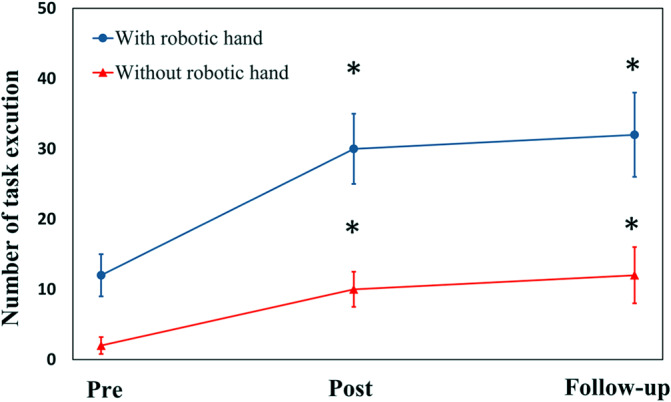
Changes of task execution numbers for evaluation trials, with and without wearing a soft robotic hand (mean value with standard deviations). The significant difference is indicated by “*” (independent t-test, p < .05).

Figure 6 depicts the variations in the number of tasks observed completed during the evaluation trial for all seven stroke subjects. The findings indicate a notable increase in execution numbers, even without wearing the soft robotic hand. This increase signifies the improvement of hand-motor functions following the soft robotic hand training. Furthermore, it is noteworthy that this effect persisted for at least 3 months after completing the training.

## V. Conclusions

This study introduces an interactive soft robotic hand system for task-oriented hand-functional stroke rehabilitation. The soft robotic hand could interact with the interactive task board and achieve different hand-functional assistance modes. The clinical outcomes following 20 training sessions involving seven chronic stroke subjects are reported. The soft robotic hand demonstrates practical assistance for stroke patients, enabling them to perform various hand-functional grasping actions and extend their spastic hand. The preliminary data indicates notable improvements in motor functions of the hand and upper limbs after the 20 training sessions, and this effect could persist for at least 3 months after training. However, future clinical studies will require a larger sample size to validate these findings further and explore the full potential of this hand-task training system in stroke rehabilitation.

## Data Availability

The data used to support the findings of the study are included in the article.

## References

[r1] Basteris A, Nijenhuis SM, Stienen AH, Buurke JH, Prange GB and Amirabdollahian F (2014) Training modalities in robot-mediated upper limb rehabilitation in stroke: a framework for classification based on a systematic review. Journal of NeuroEngineering and Rehabilitation 11, 111. 25012864 10.1186/1743-0003-11-111 PMC4108977

[r2] Bützer T, Lambercy O, Arata J and Gassert R (2021) Fully wearable actuated soft exoskeleton for grasping assistance in everyday activities. Soft Robotics 8(2), 128–143. 32552422 10.1089/soro.2019.0135

[r3] Dellon B and Matsuoka Y (2007) Prosthetics, exoskeletons, and rehabilitation - Now and for the future. IEEE Robotics & Automation Magazine 14(1), 30–34.

[r4] Duncan PW, Bode RK, Min Lai S, Perera S, and Glycine Antagonist in Neuroprotection Americans Investigators (2003) Rasch analysis of a new stroke-specific outcome scale: the Stroke Impact Scale. Archives of Physical Medicine and Rehabilitation 84(7), 950–963. 12881816 10.1016/s0003-9993(03)00035-2

[r5] Heung HL, Tang ZQ, Shi XQ, Tong KY and Li Z (2020) Soft rehabilitation actuator with integrated post-stroke finger spasticity evaluation. Frontiers in Bioengineering and Biotechnology 8, 111. 32181247 10.3389/fbioe.2020.00111 PMC7059754

[r6] Heung KHL, Tong RKY, Lau ATH and Li Z (2019) Robotic glove with soft-elastic composite actuators for assisting activities of daily living. Soft Robotics 6(2), 289–304. 30874489 10.1089/soro.2017.0125

[r7] Ho NSK et al. (2011) An EMG-driven exoskeleton hand robotic training device on chronic stroke subjects task training system for stroke rehabilitation. IEEE International Conference on Rehabilitation Robotics 1–5. 10.1109/ICORR.2011.5975340 22275545

[r8] Hu XL, Tong KY, Song R, Zheng XJ and Leung WWF (2009) A comparison between electromyography-driven robot and passive motion device on wrist rehabilitation for chronic stroke. Neurorehabilitation and Neural Repair 23(8), 837–846. 19531605 10.1177/1545968309338191

[r9] Jonkman EJ, de Weerd AW and Vrijens NLH (1998) Quality of life after a first ischemic stroke - Long-term developments and correlations with changes in neurological deficit, mood and cognitive impairment. Acta Neurologica Scandinavica 98(3), 169–175. 9786613 10.1111/j.1600-0404.1998.tb07289.x

[r10] Kwakkel G, Kollen BJ, van der Grond J and Prevo AJH (2003) Probability of regaining dexterity in the flaccid upper limb - Impact of severity of paresis and time since onset in acute stroke. Stroke 34(9), 2181–2186. 12907818 10.1161/01.STR.0000087172.16305.CD

[r11] Leibovic SJ and Bowers WH (1994) Anatomy of the proximal interphalangeal joint. Hand Clinics 10(2), 169–178. 8040195

[r12] Maciejasz P, Eschweiler J, Gerlach-Hahn K, Jansen-Troy A and Leonhardt S (2014) A survey on robotic devices for upper limb rehabilitation. Journal of NeuroEngineering and Rehabilitation 11, 3. 24401110 10.1186/1743-0003-11-3 PMC4029785

[r13] Peters M, Mackenzie K and Bryden P (2002) Finger length and distal finger extent patterns in humans. American Journal of Biological Anthropology 117(3), 209–217. 10.1002/ajpa.10029 11842400

[r14] Polygerinos P, Wang Z, Galloway KC, Wood RJ and Walsh CJ (2015) Soft robotic glove for combined assistance and at-home rehabilitation. Robotics and Autonomous Systems 73, 135–143.

[r15] Shi XQ, Heung HL, Tang ZQ, Li Z and Tong KY (2021) Effects of a soft robotic hand for hand rehabilitation in chronic stroke survivors. Journal of Stroke and Cerebrovascular Diseases 30(7) 105812. 33895427 10.1016/j.jstrokecerebrovasdis.2021.105812

[r16] Shi XQ, Heung HL, Tang ZQ, Tong KY and Li Z (2020) Verification of finger joint stiffness estimation method with soft robotic actuator. Frontiers in Bioengineering and Biotechnology 8, 592637. 33392166 10.3389/fbioe.2020.592637 PMC7775510

[r17] Song R, Tong KY, Hu XL and Li L (2008) Assistive control system using continuous myoelectric signal in robot-aided arm training for patients after stroke. IEEE Transactions on Neural Systems and Rehabilitation 16(4), 371–379. 10.1109/TNSRE.2008.926707 18701384

[r18] Susanto EA, Tong RKY, Ockenfeld C and Ho NSK (2015) Efficacy of robot-assisted fingers training in chronic stroke survivors: a pilot randomized-controlled trial. Journal of Neuroengineering and Rehabilitation 12, 1–9. 25906983 10.1186/s12984-015-0033-5 PMC4422529

[r19] Tang ZQ, Heung HL, Shi XQ, Tong RKY and Li Z (2022) Probabilistic model-based learning control of a soft pneumatic glove for hand rehabilitation. IEEE Transactions on Biomedical Engineering 69(2), 1016–1028. 34516370 10.1109/TBME.2021.3111891

[r20] Tang ZQ, Heung HL, Tong KY and Li Z (2021) Model-based online learning and adaptive control for a “human-wearable soft robot” integrated system. International Journal of Robotics Research 40(1), 256–276.

[r21] Timmermans AA, Spooren AI, Kingma H and Seelen HA (2010) Influence of task-oriented training content on skilled arm-hand performance in stroke: a systematic review. Neurorehabilitation and Neural Repair 24(9), 858–870. 20921325 10.1177/1545968310368963

[r22] Tong KY et al. (2010) An intention driven hand functions task training robotic system. IEEE Engineering in Medicine and Biology 3406–3409. 10.1109/IEMBS.2010.5627930 21097247

[r23] Wolbrecht ET, Rowe JB, Chan V, Ingemanson ML, Cramer SC and Reinkensmeyer DJ (2018) Finger strength, individuation, and their interaction: relationship to hand function and corticospinal tract injury after stroke. Clinical Neurophysiology 129(4), 797–808. 29453171 10.1016/j.clinph.2018.01.057 PMC5856636

